# Effect of Adding Bovine Skin Gelatin Hydrolysates on Antioxidant Properties, Texture, and Color in Chicken Meat Processing

**DOI:** 10.3390/foods12071496

**Published:** 2023-04-02

**Authors:** Suleivys M. Nuñez, Constanza Cárdenas, Pedro Valencia, Marlene Pinto, Javier Silva, Ernesto Pino-Cortés, Sergio Almonacid

**Affiliations:** 1 Escuela de Ingeniería Química, Pontificia Universidad Católica de Valparaíso, Valparaíso 2340025, Chile; 2 Núcleo de Biotecnología Curauma (NBC), Pontificia Universidad Católica de Valparaíso, Valparaíso 2373223, Chile; 3 Departamento de Ingeniería Química y Ambiental, Universidad Técnica Federico Santa María, Valparaíso 2390123, Chile

**Keywords:** bovine skin gelatin hydrolysates, antioxidant activity, texture, color, chicken meat

## Abstract

(1) Background: Phosphates are used in the food industry to improve water retention and product quality. However, when consumed in excess, they can be harmful to health. Instead, bovine skin gelatin hydrolysates present health benefits such as being a rejuvenating agent, stimulating collagen production, and improving food quality, in addition to being a source of protein. The effect of the addition of bovine skin gelatin hydrolysates on the texture and color of thermally processed chicken meat (boiled type) and antioxidant activity was evaluated. (2) Methods: Hydrolysates were prepared with subtilisin with the degree of hydrolysis being 6.57 and 13.14%, which were obtained from our previous study. (3) Results: The hydrolysates improved the firmness of the meat matrix compared to the control. Additionally, the hydrolysate with a 13.14% degree of hydrolysis reached the same firmness (*p* > 0.05) as the commercial ingredient sodium tripolyphosphate at its maximum limit allowed in the food industry when it was applied at 5% (*w*/*w* meat) in the meat matrix, improving firmness over the control by 63%. Furthermore, both hydrolysates reached a similar color difference to sodium tripolyphosphate at its maximum allowed limit when applied at a concentration of 2% (*w*/*w* meat). Additionally, it was found that these hydrolysates obtained the same antioxidant activity as sodium tripolyphosphate, capturing free radicals at 10%. (4) Conclusion: The findings of this study suggest that bovine skin gelatin hydrolysates can be applied as an ingredient with functional properties, being an alternative to phosphates to improve the quality of meat products.

## 1. Introduction

At present, the demand for healthy food is a latent and fundamental problem for the food industry [1]. In recent years, there has been an excessive increase in the human consumption of food products containing artificial additives [2]. An example of these additives is the phosphates incorporated in many food products, including chicken meat, the intake of which is estimated to have increased dramatically in recent years [3]. 

In the food industry, artificial additives are common and must be included in the labeling [4]. Although they are considered safe for health, sometimes public perception is altered due to these additives’ chemical names, causing rejection [5]. In this sense, the safety of polyphosphates as food additives was re-evaluated by a scientific panel of experts [6]. The panel concluded that an ADI (acceptable daily intake) of 40 mg/kg-person per day, expressed as phosphorus, protects healthy adults. This dose is less than the doses at which relevant adverse effects have been reported in short- and long-term studies in humans. However, this ADI does not apply to humans with a moderate to severe kidney function reduction [6]. Epidemiological, in vitro, in vivo, and clinical studies have reported that phosphates can cause disturbance in the human organism when consumed excessively. Studies have shown a high correlation between organ calcification and phosphate concentration in patients with kidney disease and a correlation between cardiovascular disease and blood with high phosphate levels in healthy people [7,8]. Therefore, finding natural ingredients that can replace these additives is an essential task in this context.

In recent years, chicken meat production has increased considerably, surpassing beef, thanks to its versatility, lower cost than other meats, and acceptance in all religions [9,10]. The main quality problems in chicken meat include lipid oxidation problems, pink color in uncured cooked breast, texture, and food safety. Research and development to ensure the safety and quality of cooked and raw chicken meat using new processing technologies is currently a problem for the industry [11].

One of the main additives used in the food industry is phosphates. Although phosphates are not natural preservatives, they have a slight antioxidant effect and are used in various processed foods to increase emulsion stability and reduce water loss when the product is prepared for consumption [12,13]. This allows for improving properties such as the texture, the uniformity of the final product, cooking performance, and color fixation [12]. These properties are of decisive importance since they are closely related to improving sensory aspects [13]. Additionally (and not least), weight loss reduction in meat products is linked to economic benefits. In this context, the water-holding capacity and other functional properties of muscle proteins can be improved by adding protein hydrolysates, improving the mentioned parameters [13,14].

Collagen is a protein that has been used in multiple industrial applications, including the food industry, mainly in the meat industry, due to its biological compatibility and low allergenicity due to its ability to bind water [15]. Collagen and its fractions (gelatin) play an essential role in the human diet, having a considerable content of essential amino acids, which also prevent joint diseases [16]. Furthermore, these products constitute a source of animal protein due to the significant amounts of nutritional fibers [17,18]. Adding collagen and its derivatives in meat products contributes to the biological value and improvement of the sensory and technological properties of the products consumed [19]. This protein has been applied to meat products to increase gelling capacity, water retention, and protein content [20]. 

Gelatin is a soluble, partially hydrolyzed protein mixture derived primarily from collagen (the cooked form of collagen) [21]. This protein mix retains more water than collagen, because it has exposed active groups that react with water through hydrogen bonding [19]. As a result, gelatin is commercially available, inexpensive, and of food grade [22].

One study analyzed the effect of adding duck leg collagen on the physicochemical properties of surimi. In that study, good results were obtained in the folding tests, gel resistance, water-holding capacity (WHC), texture profile analysis, and color measurement [23]. A significant improvement in the quality of sardine surimi was obtained. A previous study evaluated the WHC of bovine skin gelatin hydrolysates obtained via enzymatic hydrolysis with subtilisin in a thermally processed chicken meat model. The bovine skin gelatin with which the hydrolysates were produced had 86.77% protein, 10% moisture, 2.25% carbohydrates, 0.7% ash, and 0.28% lipids. The hydrolysates obtained with Alcalase presented degrees of hydrolysis of 6.57 and 13.14%. 

We compared it with the commercial additive sodium tripolyphosphate (STPP), obtaining good results [24]. In said study, the hydrolysates reached the same WHC as the commercial ingredient STPP applied at the highest concentration allowed in the industry (0.5% *w*/*w*) when applied at 3 and 2.5% (*w*/*w*) in the meat matrix. These hydrolysates can replace STPP as a WHC agent and reduce the cooking loss by 33% and 42%, respectively, when applied at 5% *w*/*w*, which was the highest concentration used in that study. However, for these hydrolysates to be a suitable substitute for polyphosphates, in addition to retaining water, they must improve the firmness of the meat matrix. Therefore, color and antioxidant activity are essential properties to consider; although phosphates are not natural preservatives, they have a slight antioxidant effect [12,13]. This could significantly improve the efficiency of the water retention effect and consequently impact the meat and ingredient industry. Therefore, this study aimed to evaluate the antioxidant activity, texture, and color of bovine skin gelatin hydrolysates in a thermally processed chicken meat model. 

## 2. Materials and Methods

### 2.1. Materials

The principal materials used in this study were bovine skin gelatin hydrolysates, namely, BSGH1 and BSGH2, with 6.57 and 13.14% DH (degrees of hydrolysis), respectively, acquired from a previous study from bovine skin gelatin (BSG) provided by Rousselot (Amparo, Sao Paulo, Brazil) [24]. These hydrolysates were obtained with the enzyme subtilisin from the commercial preparation of 2.4 L of Alcalase supplied by Novozymes (Bagsvaerd, Copenhague, Denmark). The hydrolysis conditions were a pH of 8, 50 °C, and an enzyme/substrate ratio of 1/4. The hydrolysis reaction times were 15 and 60 min. The skinless chicken (unmarinated commercial product) breast meat was acquired from a local meat processing company, Agrosuper (Viña del Mar, Valparaíso, Chile). Analytical-grade reagents were used in this study for all experiments.

### 2.2. Sample Preparation

The samples were elaborated with the same proportions as for 1 g of meat, according to [24]. The sample was a mixture containing 15 g of ground chicken meat at room temperature in an Oster blender and 2.7 g of distilled water. BSGH1 and BSGH2 were added to the chicken meat at concentrations of 1, 2, 3, 4, and 5% (*w*/*w* meat) and mixed. Positive controls with commercial STPP added at all five concentrations and a negative control without BSGHs were prepared. The samples were stored in 50 mL falcon tubes (JET BIOFIL^®^, Guangzhou, China), refrigerated for 1 h, and finally subjected to a heat treatment.

### 2.3. Thermal Treatment

The meat sample was incubated in a heat bath at 80 °C until reaching a temperature of 72 °C inside in order to guarantee food safety. The time required to reach that temperature was determined. For this aim, a thermocouple (Type T, #TEF-22-S, TechniCAL LLC) was placed in the center of the sample. After this, the samples were cooled with cold water. TracerDAQ^®^ software (Norwalk, CT, USA) was used for data recording [24].

### 2.4. Texture Determination of Meat Samples

Texture analysis quantifies the food’s mechanical behavior by subjecting it to a defined force and measuring the resulting deformation curve. A certain number of parameters are obtained from the force vs. time curve analysis, such as adhesiveness, cohesiveness, firmness, elasticity, and relaxation time [25]. Texture analysis of chicken meat processed with BSGH1, BSGH2, or STPP at concentrations of 0–5% (*w*/*w* meat) was performed on a previously calibrated CT3 texture analyzer (Brookfield, Middleboro, MA, USA), equipped with a 12.7 mm diameter round-nose cylindrical plunger under the operating conditions specified in Table 1. Measurement data are presented as “firmness”, which is defined as the force (in Newtons) required to achieve 30% deformation in compression tests. This can be correlated to a measure of stiffness (force/deformation) at a specific degree of deformation, which the consumer would perceive as “firmness” [26]. 

### 2.5. Color Determination of Meat Samples

Color measurements were carried out with a CR-400 colorimeter with an aperture opening size of 8 mm, observer angle of 0<°, and illuminant-type C, D65 (Chroma Meter; Konica Minolta Sensing Inc. Osaka, Japan) using the L*, a*, b* color parameter system. The L* parameter shows the brightness, the a* parameter represents the redness, and the b* parameter represents the yellowness. Measurements were carried out in triplicate in the center of each sample after heat treatment. The average of three colorimeter readings was taken as the result of one measurement. The color difference between 2 objects is determined by calculating the ΔE* value (Equation (1)). This corresponds to the “difference in sensation” perceived by the exposure of two colors.

(1) $${\Delta E}^{*}=\sqrt{{\Delta L}^{*2}+{\Delta a}^{*2}+{\Delta b}^{*2}}$$

where Δ*L*, Δ*a*, and Δ*b* are the differences in parameters *L*, *a*, and *b* between both samples to be compared; ΔE* must have a value greater than 3 to have an appreciable color difference [27]. For the color differences, each datum of the ΔE* value was calculated concerning the sample’s average compared with the control (0% ingredient).

### 2.6. Antioxidant Activity of BSGHs

The method used to determine the antioxidant capacity is based on the capture of free radicals. The antioxidant activity is detected by a free radical scavenging reaction with 2,2-Diphenyl-1-picrylhydrazyl (DPPH). This reagent with a violet color changes to a colorless substance when reduced due to the effect of an antioxidant compound [28]. The scavenging effect on the DPPH radical can be measured as the ratio between the absorbances, read as follows:

(2) $$Scavenging\;effect\left(\%\right)=\left[\frac{Abs\;control-(Abs\;sample-Abs\;blank)}{Abs\;control}\right]\cdot 100$$

where *Abs sample* is the absorbance of BSGHs with DPPH, *Abs control* is the absorbance of DPPH without hydrolysates, and *Abs blank* is the absorbance of BSGHs without DPPH. DPPH with ethanol was used as the control, and water with hydrolysates was the blank. 

DPPH is a solidly marketed reagent. For the preparation of the solution, it was dissolved in analytical-grade ethanol (99%) at a concentration of 30 mg/L. The samples of the BSGHs were prepared at variable concentrations (1, 2, and 3 mg/mL) in distilled water. The procedure was based on that described by [29]; with modifications, 140 μL of the sample was mixed with 1.4 mL of DPPH solution in an Eppendorf tube, with three replicates for each sample. Subsequently, they were left on an orbital shaker at 25 °C for 1 h. Finally, the absorbance of the samples was read at 520 nm.

### 2.7. Statistical Analysis

The data are presented as the mean value ± standard deviation of three replicates. For the statistical analysis, analysis of variance (ANOVA) was used. The LSD Fisher test was used to perform multiple comparisons of the data, considering a value of *p* ≤ 0.05 significant using the InfoStat/L software. The effect of each treatment was compared (“treatment” meaning each ingredient at each concentration).

## 3. Results and Discussion

### 3.1. Thermal Treatment

**Figure 1:**
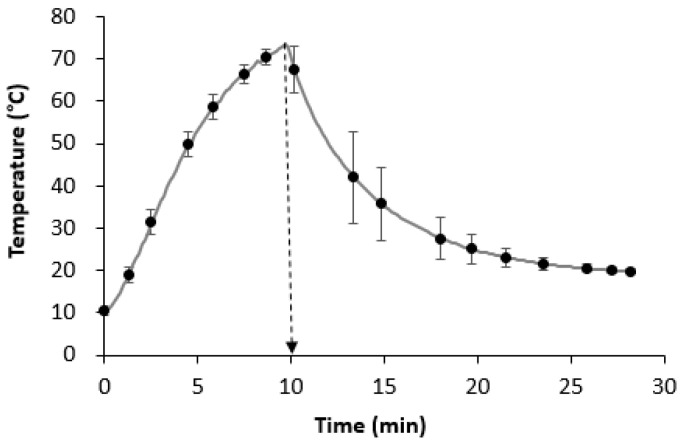
Temperature behavior over time during heat treatment of chicken meat samples cooked at 80 °C. The values are presented as the mean ± standard deviation of three replicates. The dashed arrow indicates the time needed to reach the temperature of 72 °C inside the sample.

In Figure 1, the behavior of temperature vs. time during the heat treatment of the samples is shown. As can be seen, after 10 min, the temperature of 72 °C was reached inside the sample. Therefore, this time was considered for the heat treatment of all samples in this study.

### 3.2. Texture Determination of Meat Samples

**Figure 2:**
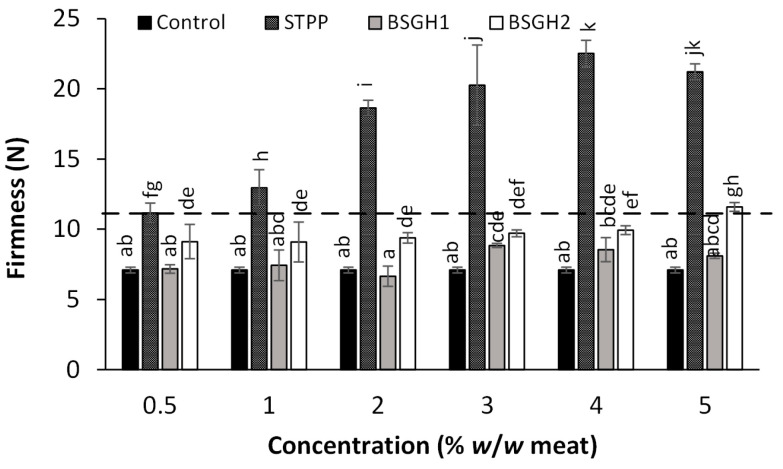
Effect of BSGH1, BSGH2, or STPP on the “firmness” (force in Newtons for 30% deformation) of thermally processed chicken meat. The values are presented as the mean ± standard deviation of three replicates. Different letters indicate significant differences (*p* ≤ 0.05). The dashed line represents the firmness of STPP at the maximum concentration allowed in the industry (0.5% *w*/*w* meat).

The results from the texture measurements on chicken meat samples processed with BSGH1, BSGH2, or STPP are summarized in Figure 2. The addition of any hydrolysates or STPP increased the meat matrix’s firmness compared to the control. This means that the samples containing additives had greater firmness (*p* ≤ 0.05), another desirable attribute for consumer acceptance [26]. 

In the case of STPP, this improvement was dependent on the concentration of the ingredient; at concentrations over 3% (*w*/*w* meat), no differences in firmness were observed (*p* > 0.05), which correlates with the WHC results obtained in our previous study [24] for this ingredient. BSGH1 improved the firmness of the meat matrix when applied at a concentration of 3% (*w*/*w* meat) (*p* ≤ 0.05), while BSGH2 reached the highest firmness (*p* ≤ 0.05) when applied at a concentration of 5% (*w*/*w* meat). The maximum permitted limit of STPP in the food industry is 0.5% (*w*/*w* meat) [13]; although BSGH1 improved firmness concerning the control, it was no better than STPP at its maximum industry-allowed limit. In contrast, BSGH2 reached the same firmness (*p* > 0.05) as STPP at its maximum allowed limit when it was applied at 5% (*w*/*w* meat) in the meat matrix, improving firmness over the control by 63%. Furthermore, the firmness of all the ingredients used was strongly correlated with the cooking loss obtained in our previous study [24], obtaining Pearson’s correlation values of −0.73, −0.93, and −0.82 for the BSGH1, BSGH2, and STPP, respectively. 

The results shown in this study are in accordance with what was shown by [20]. It is shown that WHC and texture were improved in sausages when they were enriched with a high content of hydrolyzed collagen (50 and 75% (*w*/*w*)), obtaining higher values of cut resistance, which indicates that the addition of collagen provides greater resistance to the product [20]. This effect is explained in the article by [30], where it is reported that when the protein matrix chemically retains water, swelling occurs; therefore, the collagen fibers confer cohesion and texture on the dough, contributing to the firmness of the final product. Similar results were obtained in another study conducted by [26], where the WHC, texture, and color of samples of salmon fillets processed with different combinations of protein hydrolysates from salmon waste and salt were evaluated. The authors attributed the increased firmness in the fillets to a probable reflection of more significant cellular turgor pressure achieved by the higher WHC attributed to adding salt or hydrolysates [26]. The WHC of the hydrolysates is attributed to the fact that more active polar groups are exposed during enzymatic hydrolysis and interact with water through hydrogen bonds. In addition, more hydrophilic peptides are obtained by generating terminal carbonyl and amino groups, contributing to water retention [14,31].

### 3.3. Color Determination of Meat Samples

The values of the color parameters (L*, a*, and b*) and each ΔE* value, measured in the cooked chicken meat samples with different concentrations (1–5% *w*/*w* meat) of each ingredient (BSGH1, BSGH2, or STPP), are shown in Table 2, Table 3, Table 4 and Table 5.

Color analysis of the meat samples showed that the luminosity values (L*) differed significantly with the concentration of each ingredient used (*p* ≤ 0.05), since the L* values decreased as the ingredient concentration increased (Table 2). This decrease in L* values could be related to an increase in the WHC of meat caused by these additives, according to our previous study [24]. The same behavior was observed by [32], where the authors evaluated the effect of STPP, NaCl, and pH on the color properties of pork meat.

Studies have shown that salt alters the osmotic balance of the tissue. When salt is added to minced meat, it penetrates its structure, changes the myofilaments’ electrostatic charges, and affects the isoelectric point of proteins, increasing the WHC [33,34]. This increase in the WHC reduces water availability on the surface of the meat, thus reducing the values of light reflection and brightness. Other studies on the process of producing meat products (cooked and dry-cured) have found that both the addition of salt and its diffusion through the muscles reduce brightness [35,36]. 

Redness (a*) and yellowness (b*) did not show much difference with different ingredient concentrations (*p* > 0.05) and did not follow a pattern (Table 3 and Table 4). Similar results were also observed by [32]. An increase in b* values has been reported in studies of cooked meat products (containing phosphates in their formulation), an effect that has been attributed to two factors: the cooking process and oxidation. However, in dry-cured products and fresh meat, no reference to an effect of phosphate on the parameter b* has been found [32]. 

The color difference in the meat samples measured as ΔE* (Table 5) depended on the concentration of each ingredient used. It is reported that ΔE* must have a value greater than 3 to represent an appreciable color difference [27]. In this case, the color differences were appreciable (*p* ≤ 0.05) for STPP when applied at 1% (*w*/*w* meat) and 3% (*w*/*w* meat) to BSGH1 and BSGH2. These hydrolysates reached similar values of ΔE* (*p* > 0.05) to STPP applied at the maximum concentration allowed in the food industry when they were applied at 2% (*w*/*w* meat) in the meat matrix. 

### 3.4. Antioxidant Activity of BSGHs

**Figure 3:**
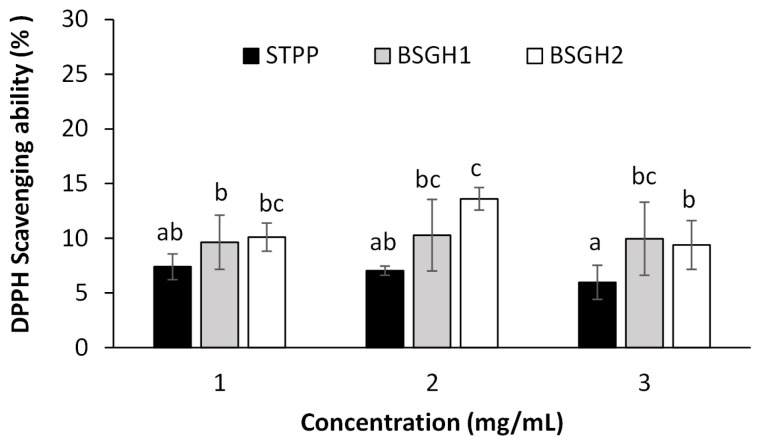
DPPH radical scavenging ability of BSGH1, BSGH2, and STPP at different concentrations. The values are expressed as the mean ± standard deviation of three replicates. Different letters indicate significant differences (*p* ≤ 0.05).

The DPPH-scavenging ability of BSGH1, BSGH2, and STPP is shown in Figure 3. Both BSGHs and STPP removed DPPH radicals by about 10% (Figure 3). The DPPH radical scavenging activities of all samples were not influenced by the concentration. No significant differences (*p* > 0.05) were observed between the hydrolysate samples and STPP, suggesting that these hydrolysates may adequately replace STPP. Although STPPs are not natural preservatives, they have a slight antioxidant effect [12,13], as seen in this study. On the other hand, it has been shown that antioxidant activity is among the broad properties of gelatin hydrolysates [37,38,39].

Studies have obtained antioxidant activity values below 20% when hydrolyzing bovine casein with trypsin alone and combined trypsin and pepsin [40]. The hydrolysate obtained with trypsin prepared over 4 h of reaction showed the highest antioxidant activity (13.7%) in this study. The antioxidant activity presented by hydrolysates can be attributed, in part, to the structural changes in proteins since enzymatic hydrolysis promotes the opening and exposure of residues of active amino acids capable of reacting with oxidants [41]. It has even been reported that excessive hydrolysis of proteins can decrease the antioxidant activity [42,43], since it leads to an increase in free amino acids, which are not good antioxidants on their own, and the size of peptides. Therefore, the physical and chemical properties conferred by the amino acid sequence are necessary, principally for the stability of peptide radicals that do not propagate or initiate further oxidative reactions [44]. Additionally, it is stated that some amino acids present in peptides, such as Lys, Tyr, and His, have antioxidant properties. Furthermore, essential amino acids can chelate metal ions, and Cys is a proton donor thanks to its thiol group. Val and Leu have been shown to exert antioxidant properties at the N-terminus of a peptide, and Tyr and Trp are found in the C-terminal sequence [45]. 

## 4. Conclusions

In conclusion, we found that bovine skin gelatin hydrolysates can achieve the same color difference, firmness, and antioxidant activity as the commercial ingredient STPP at its maximum concentration allowed in the food industry. Therefore, these enzymatically prepared hydrolysate compositions reflect their functional properties and their potential application in the food industry to replace STPP. However, additional studies are needed to identify the peptide fractions responsible for the antioxidant activity of these ingredients, as well as the fractions responsible for the WHC, and to delve into the effect of the WHC on the luminosity and texture of the meat matrix. It would also be interesting to evaluate the parameters studied in this work with other meat matrices. 

## Figures and Tables

**Table 1:** Operating conditions used with the Brookfield texture analyzer, Middleboro.

Parameter	Value
Type of sample	Cylinder
Sample length	40 mm
Sample diameter	26.2 mm
Charge	25,000 g
Test speed	1 mm/s
Activation charge	0.5 N
% deformation	30
Test	Compression
Probe	TA 18

**Table 2:** Average values of the color parameter L* for STPP, BSGH1, and BSGH2. Results are presented as the average of three replicates ± standard deviation. Different letters indicate significant differences (*p* ≤ 0.05).

Concentration (% *w*/*w* Meat)	Parameter L*		
	STPP	BSGH1	BSGH2
Control	86.31 ± 0.99 ^a^	86.31 ± 0.99 ^a^	86.31 ± 0.99 ^a^
0.5	84.96 ± 0.16 ^b^	85.42 ± 0.31 ^ab^	85.81 ± 0.09 ^ab^
1	83.87 ± 0.30 ^c^	85.18 ± 0.15 ^b^	85.20 ± 0.19 ^b^
2	79.89 ± 1.12 ^h^	83.81 ± 0.08 ^c^	83.07 ± 0.50 ^cd^
3	76.63 ± 0.55 ^i^	82.37 ± 0.12 ^de^	81.35 ± 0.21 ^fg^
4	76.17 ± 0.64 ^i^	81.81 ± 0.78 ^ef^	80.60 ± 0.74 ^g^
5	75.23 ± 0.67 ^j^	80.74 ± 0.57 ^g^	78.02 ± 0.58 ^h^

**Table 3:** Average values of the color parameter a* for STPP, BSGH1, and BSGH2. Results are presented as the average of three replicates ± standard deviation. Different letters indicate significant differences (*p* ≤ 0.05).

Concentration (% *w*/*w* Meat)	Parameter a*		
	STPP	BSGH1	BSGH2
Control	1.49 ± 0.86 ^de^	1.49 ± 0.86 ^de^	1.49 ± 0.86 ^de^
0.5	1.16 ± 0.24 ^cd^	2.14 ± 0.11 ^hi^	1.63 ± 0.20 ^ef^
1	0.88 ± 0.28 ^bc^	2.10 ± 0.27 ^hi^	1.67 ± 0.24 ^efg^
2	0.59 ± 0.36 ^ab^	2.07 ± 0.10 ^ghi^	1.81 ± 0.27 ^efgh^
3	0.30 ± 0.15 ^a^	2.24 ± 0.05 ^i^	2.18 ± 0.13 ^hi^
4	0.43 ± 0.09 ^a^	1.99 ± 0.34 ^fghi^	2.10 ± 0.29 ^hi^
5	0.19 ± 0.08 ^a^	2.16 ± 0.17 ^hi^	2.09 ± 0.17 ^ghi^

**Table 4:** Average values of the color parameter b* for STPP, BSGH1, and BSGH2. Results are presented as the average of three replicates ± standard deviation. Different letters indicate significant differences (*p* ≤ 0.05).

Concentration (% *w*/*w* Meat)	Parameter b*		
	STPP	BSGH1	BSGH2
Control	12.80 ± 0.90 ^abcd^	12.80 ± 0.90 ^abcd^	12.80 ± 0.90 ^abcd^
0.5	13.38 ± 0.15 ^a^	12.04 ± 0.37 ^d^	12.02 ± 0.31 ^d^
1	12.81 ± 0.63 ^abcd^	12.50 ± 0.14 ^bcd^	12.33 ± 0.11 ^cd^
2	10.20 ± 1.51 ^e^	13.05 ± 0.11 ^abc^	13.09 ± 0.09 ^abc^
3	9.49 ± 0.27 ^e^	13.34 ± 0.18 ^ab^	13.46 ± 0.31 ^a^
4	9.81 ± 1.07 ^e^	13.36 ± 0.16 ^ab^	13.54 ± 0.21 ^a^
5	10.05 ± 0.19 ^e^	13.39 ± 0.45 ^a^	13.03 ± 0.09 ^abc^

**Table 5:** Color differences (ΔE*) in cooked chicken meat samples with different BSGH1, BSGH2, or STPP concentrations. Results are presented as the average of three replicates ± standard deviation. Different letters indicate significant differences (*p* ≤ 0.05).

Concentration (% *w*/*w* Meat)	ΔE*		
	STPP	BSGH1	BSGH2
0.5	2.08 ± 0.19 ^b^	0.45 ± 0.15 ^a^	0.61 ± 0.06 ^a^
1	3.24 ± 0.39 ^cd^	0.60 ± 0.14 ^a^	0.65 ± 0.18 ^a^
2	8.78 ± 1.60 ^h^	2.01 ± 0.07 ^b^	2.73 ± 0.49 ^bc^
3	11.13 ± 0.50 ^i^	3.45 ± 0.14 ^cd^	4.46 ± 0.29 ^ef^
4	11.47 ± 0.65 ^Ij^	4.01 ± 0.71 ^de^	5.21 ± 0.65 ^f^
5	12.27 ± 0.67 ^j^	5.03 ± 0.61 ^f^	7.64 ± 0.57 ^g^

## Data Availability

Data available on request due to restrictions e.g. privacy or ethical: The data presented in this study are available on request from the corresponding author.
